# Enhanced Optical Limiting of Gold Nanoparticles/Porous Carbon Nanocomposites

**DOI:** 10.3390/ma17133079

**Published:** 2024-06-22

**Authors:** Bo Gao, Xuhui Zhao, Lijiao Yang, Lihe Yan, Tao Lin, Jinhai Si

**Affiliations:** 1 Department of Electronic Engineering, Xi’an University of Technology, Xi’an 710048, China; 2210320011@stu.xaut.edu.cn (X.Z.); 2210320015@stu.xaut.edu.cn (L.Y.); lintao@xaut.edu.cn (T.L.); 2 Laboratory of Photonics Technology for Information, School of Electronic Science and Engineering, Xi’an Jiaotong University, No. 28, Xianning West Road, Xi’an 710049, China; liheyan@mail.xjtu.edu.cn (L.Y.); jinhaisi@mail.xjtu.edu.cn (J.S.)

**Keywords:** gold nanoparticles/porous carbon nanocomposites, optical limiting, laser protection

## Abstract

With the wide application of laser weapons, the requirements of laser protection technology are becoming more and more strict. Therefore, it is important to find ideal optical limiting (OL) materials to protect human eyes and detectors. In this work, the nonlinear optical responses of gold nanoparticles/porous carbon (Au NPs/PC) nanocomposites prepared by the reduction method were studied using the nanosecond Z-scan technique. Compared with porous carbon, the Au NPs/PC nanocomposites show a lower damage threshold, a bigger optical limiting index and a wider absorption spectrum. The interaction between gold nanoparticles and porous carbon enhances the nonlinear scattering effect of suspended bubbles. These results indicate that Au NPs composites have potential applications in the protection of human eyes and detectors.

## 1. Introduction

With the wide application of laser application systems in scientific research activities and military weapons, the research on precise optical instruments and human eye protection technology has become increasingly important. Laser has excellent properties such as strong directivity, good monochromaticity, high coherence and high brightness. It has been widely used in civil and military fields such as optical fiber communication, laser ranging, laser radar and so on [1,2]. With the rapid development of laser application systems, the research about laser protection has become more and more significant. The optical limiting (OL) effect is a very important nonlinear optical phenomenon that protects optically sensitive optoelectronic devices and the human eyes [3]. The ideal OL materials possess the characteristics of high linear transmittance, low confinement threshold, high damage threshold, fast response speed and wide response spectrum. Over the past decades, a number of organic materials have been extensively studied as good candidates for optical limiters, including fullerene C60 [4], carbon nanotubes [5,6], graphene [7], carbon black suspension [8], carbon quantum dots [9] and carbon nanodots [10], which can strongly weaken strong incident light. However, the OL properties of organic materials come from their excited-state absorption effect, which means the optical limiting effect is only possible within a specific wavelength range determined by the molecules’ excited energy level.

Porous carbon materials are widely used in adsorption, catalysis, electrochemistry and other fields because of the characteristics of large specific surface area, strong adsorption capacity and developed pore structure [11,12,13,14]. For example, Li et al. [15] prepared porous carbon materials by the Flash Joule heating (FJH) method, which resulted in good adsorption properties; Gao et al. [13] used biomass-based porous carbon materials as electrodes for supercapacitors, showing excellent stability and cycle characteristics. In previous studies, our group has studied the optical limiting properties of porous carbon materials at 532 nm and 1064 nm. Compared with traditional optical limiting materials, porous carbon materials show an excellent broadband limiting effect and a low limiting threshold, and their optical limiting effect is mainly due to nonlinear scattering (NLS) caused by the rapid growth and expansion of plasma and suspended bubbles [16]. Gold nanoparticles have excellent electrical conductivity, chemical stability, high specific surface area, good biocompatibility and catalytic activity. The optical properties of gold nanoparticles are closely related to the size of the generated gold nanoparticles [17,18]. The gold nanoparticles are widely used in the preparation of composite materials to improve the nonlinear optical effect of materials. For example, Prabin Pradhan et al. prepared gold-modified graphene nanocomposites, which can be obtained by higher damage threshold and better optical limiting performance [19]. Porous carbon supported by gold nanoparticles are widely used in biology, electrochemistry, catalysis and other fields [18,20,21]. Although Au NPs/PC have been used for electrochemical energy storge, absorption and other fields, the nonlinear optical limiting of Au NPs/PC nanocomposites has not been reported.

In this paper, gold nanoparticles/porous carbon nanocomposites were prepared by the reduction method. We studied the OL effect of Au NPs/PC nanocomposites using the nanosecond laser Z-scan method. Compared with porous carbon, the Au NPs/PC nanocomposites show a lower damage threshold, a bigger optical limiting index, and a wider absorption spectrum.

## 2. Experiment and Sample Preparation

In this study, porous carbon materials (purchased from XFNano Materials Tech Co., Ltd., Nanjing, China) were dissolved in deionized water by stirring for 30 min and collected by centrifugation. The resulting materials were vacuum-dried at 180 °C, ground to obtain a carbon powder and dispersed. Dispersion A were prepared by adding an appropriate amount of HAuCl_4_·3H_2_O (purchased from Aladdin Reagent Company, Shanghai, China) to 20 mL of deionized water and were sonicated for 1 h at room temperature with a power of 200 W. The dispersions were made by mixing 20 mg of porous carbon powder with 40 mL of deionized water and sonication for 1 h at room temperature with a power of 200 W. Then 50 mg polyvinylpyrrolidone (purchased from Aladdin Reagent Company, Shanghai, China) was added in porous carbon dispersions and ultrasound for 10 min with a power of 200 W and stirred for 20 min; the resulting dispersion was recorded as B. Then, solution A was added to dispersion B, and the PH value was adjusted to about 3 with concentrated hydrochloric acid solution. The 0.072 mM ascorbic acid (purchased from Aladdin Reagent Company, Shanghai, China) solution was added to the mixed solution and stirred for 30 min. The product after the reaction was ultrasonically treated at room temperature for 10 min and stirred for 30 min. The product was collected by centrifugation and washed with anhydrous ethanol and centrifuged. The collected product was dissolved in dimethyl sulfoxide (DMSO) solution and stirred for 1 h at room temperature to prevent the agglomerates. The linear transmittance of the Au NPs/PC dispersions were adjusted to 70% and quartz glass was used as cell materials. The absorption spectrum of the sample was analyzed by UV-2600, (shimadzu, Hong Kong, China). The morphology of the samples was investigated by transmission electron microscopy (TEM) and field emission scanning electron microscopy (SEM) using a JEOL JEM-2100Plus (China Educational Instrument & Equipment Corp, Beijing, China) and Quanta 250FEG (FEI, Hillsborough, OR, USA). To analyze the chemical composition of samples, the X-ray photoelectron spectroscope (XPS) method was used with Thermo Fisher ESCALAB Xi+ ( Langrun International, Xi’an, China). Raman spectroscopy was performed using a Raman spectrometer laser confocal microscope (inVia Qontor, Renishaw, Stonehouse, UK).

**Figure 1:**
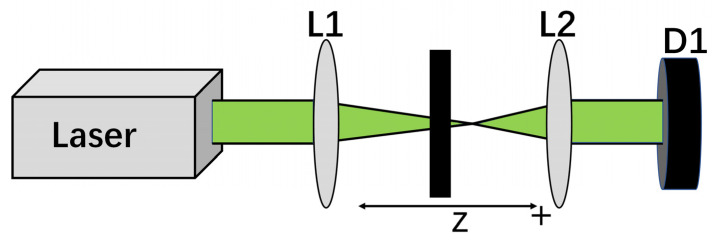
Optical path diagram of aperture Z-scan experiment.

In this study, the OL properties of Au NPs/PC nanocomposites were studied using a nanosecond aperture z-scan system. As shown in Figure 1, the nanosecond laser source Nd^3+^: YAG laser was produced by Continuum Company (West Boston, MA, USA), with a pulse repetition rate of 10 Hz, a central wavelength of 1064 nm and a pulse width of 3~7 ns. The output pulse of the laser can produce 532 nm laser pulses through the internal frequency-doubling crystal. These pulses are then focused onto the sample through a lens, L1, with a focal length of 20 cm. The beam passing through the sample is re-collimated through another lens, L2, and passes through the aperture to filter out the scattered light. The sample is fixed on a movable platform. By changing its position, the nonlinear transmittance change of the sample at different positions can be measured by using the energy meter D1.

## 3. Results and Discussion

### 3.1. Characterization Results and Analysis

**Figure 2:**
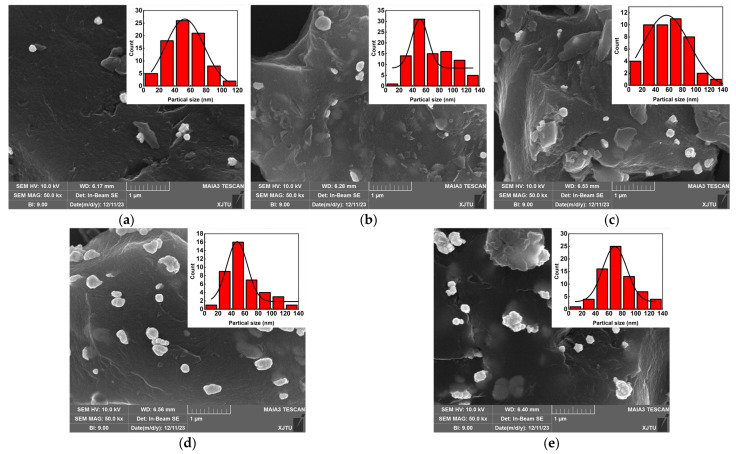
SEM images of Au NPs/PC nanocomposites prepared with different Au^3+^ concentrations (**a**) 0.01 mmol; (**b**) 0.03 mmol; (**c**) 0.06 mmol; (**d**) 0.09 mmol; (**e**) 0.12 mmol;.

To analyze the morphology of AuNPs/PC nanocomposites prepared by different Au^3+^ concentrations, SEM images were obtained. Figure 2a–e show the SEM images of Au NPs/PC nanocomposites prepared with Au^3+^ concentrations from 0.01 mmol to 0.12 mmol. The generated gold nanoparticles are well adsorbed on the surface of porous carbon materials. As the Au^3+^ concentration increased, the size of the Au NPs became larger and the concentration of gold nanoparticles increased. The upper right corner of Figure 2a–e shows the size distribution of the generated Au nanoparticles. The size of the generated Au nanoparticles is distributed between 0 nm and 140 nm. It is the uneven distribution of Au^3+^ in the dispersion during the reaction which leads to the rapid reduction of some Au^3+^ to cluster into small particles, while the remainder is attached to the original Au nucleus after reduction to continue to grow and increase in size [22]. 

**Figure 3:**
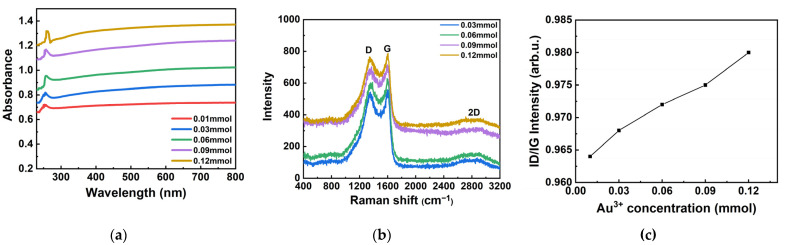
The typical characterization diagrams of Au NPs/PC composites: (**a**) UV–Vis absorption spectrum; (**b**) Raman spectrum; (**c**) *I_D_/I_G_* values.

Figure 3a shows the UV–visible absorption spectrum of Au NPs/PC composites prepared with different Au^3+^ concentrations. The strong absorption band of Au nanoparticles is assigned to the π-π* transition of the porous carbon material ligand. The absorption bands of samples are located in the ultraviolet region, indicating that the composites have good light absorption in the visible region (400 nm–700 nm) [23]. The obvious absorption peak, which corresponds to the original absorption peak of porous carbon materials, indicating that porous carbon materials have a strong absorption of light in the range of 255 nm–280 nm. With the increase in Au^3+^ concentration, the absorption peak has a very small red shift, indicating that the interaction between Au nanoparticles and porous carbon materials enhances the absorption effect of porous carbon materials. However, it increased steadily in the range of 500 nm–600 nm and did not show any absorption peak. It indicated that the surface plasmon absorption of the generated Au NPs was weak, which was caused by the attenuation of the protective agent. This situation has been reported in the relevant literature [24]. According to the Raman spectrum of the Au NPs/PC nanocomposites (Figure 3b), there are two obvious peaks: one near 1350 cm^−1^ corresponding to the D band which is attributed to disorder or defects in the carbon atoms band and 1600 cm^−1^ corresponding to the G band which is attributed to the sp^2^ in-plane vibration of carbon atoms [25,26,27], respectively. The intensity ratio of these peaks (*I_D_/I_G_*) determines the degree of graphitization in carbon materials [28,29,30]. As shown in Figure 3c, the *I_D_/I_G_* values of the Au NPs/PC nanocomposites (0.964–0.981) by the reduced method with different Au^3+^ concentrations were obtained in this work, which are significantly higher than the porous carbon (0.85). In addition, the layered graphene structure in porous carbon can be demonstrated by the characteristic 2D band near 2800 cm^−1^, which can promote the transfer of ions to reduce resistance.

**Figure 4:**
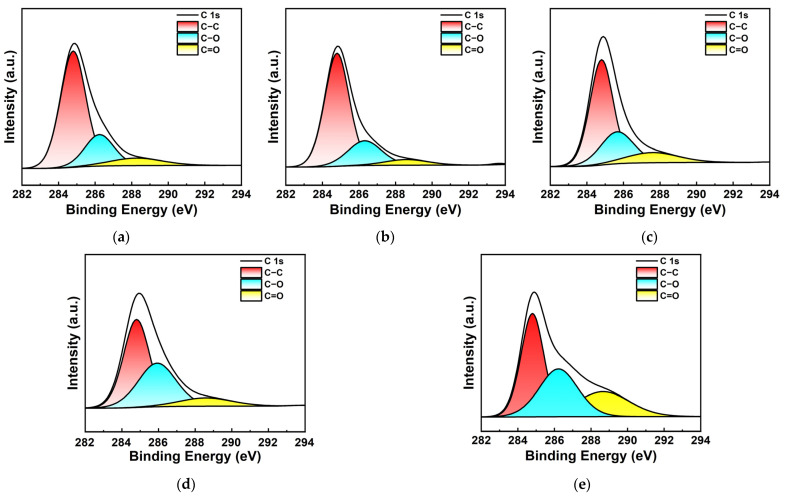
XPS spectra of C 1s of Au NPs/PC prepared with different Au^3+^ concentration of (**a**) 0.01 mmol; (**b**) 0.03 mmol; (**c**) 0.06 mmol; (**d**) 0.09 mmol; (**e**) 0.12 mmol.

Figure 4 shows the C 1s XPS spectra of Au NPs/PC nanocomposites prepared by the reduction method with different Au^3+^ concentrations. Peaks at 284.8 eV, 286.3 eV and 288.5 eV which are attributed to C−C, C−O, and C=O groups are observed in the samples [31]. 

**Figure 5:**
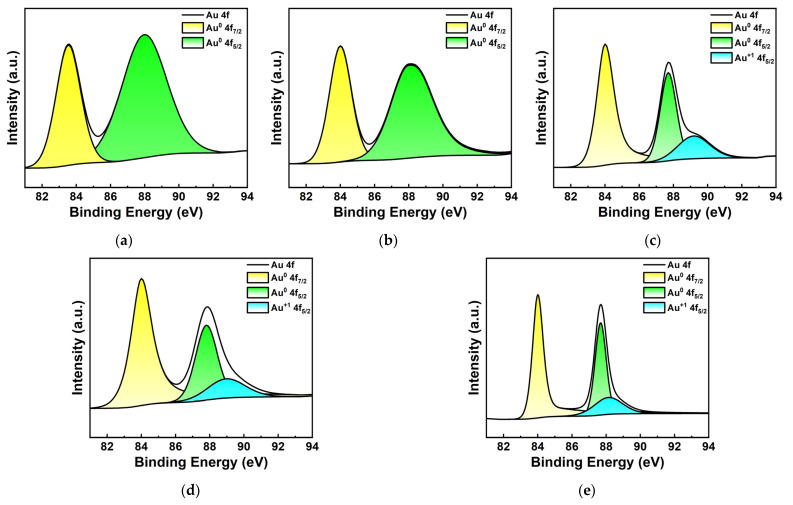
XPS spectra of Au 4f of Au NPs/PC prepared with different Au^3+^ concentration of (**a**) 0.01 mmol; (**b**) 0.03 mmol; (**c**) 0.06 mmol; (**d**) 0.09 mmol; (**e**) 0.12 mmol.

Figure 5 shows the Au 4f high-resolution XPS spectrum of Au NPs/PC nanocomposites prepared with different Au^3+^ concentrations. As shown in Figure 5a,b, when the concentrations of Au^3+^ are lower, the two absorption peaks is centered at 84 eV and 88 eV corresponding to the binding energies of Au^0^ 4f_7/2_ and Au^0^ 4f_5/2_ of Au, respectively. As shown in Figure 5c,d, with the increase in Au^3+^ concentration, Au 4f_7/2_ and Au 4f_5/2_ dissociate into two smaller absorption peaks at 83.8 eV, 87.69 eV and 89.16 eV, corresponding to the oxidation states of Au^0^ 4f_7/2_, Au^0^ 4f_5/2_ and Au^+1^ 4f_5/2_ [32,33], respectively, which were due to the incomplete reduction of the precursor [34]. In fact, the generated Au nanoparticles show high surface energy, which is another major factor in the presence of gold oxide particles. In general, the difference between the position of the characteristic peak corresponding to Au^0^ and Au^3+^ in the Au 4f XPS spectrum does not exceed 1 eV, which is difficult to distinguish in the spectrum.

### 3.2. OL Properties and Mechanisms of Au NPs/PC Nanocomposites

The nonlinear OL behavior of the Au NPs/PC nanocomposites for 532 nm is studied using the nanosecond laser z-scan technique. Au NPs/PC nanocomposite dispersion was used in the experiment. In the test, the cuvette was continuously shaken to ensure the stability of the Au NPs/PC nanocomposites dispersion. To ensure the accuracy of the experiment, we measured the samples several times below the same conditions to obtain the average value. At the same time, the z-scan signal of the quartz cuvette was measured, and the value of this part was removed in the sample signal.

**Figure 6:**
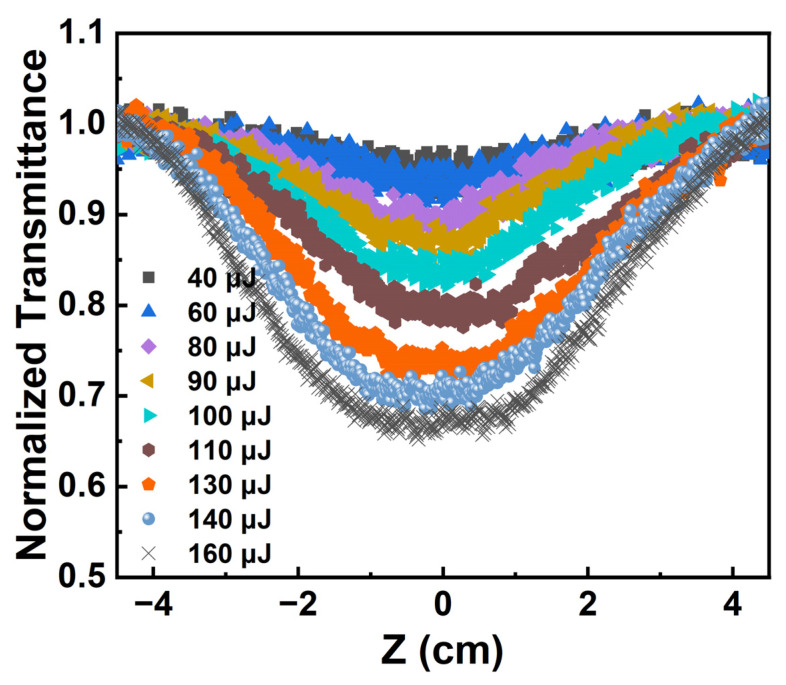
At 532 nm: Au NPs/PC nanocomposites z-scan curves as the pulse energy changes.

In order to explore the OL response intensity of the Au NPs/PC nanocomposites, as a reference, z-scan measurements of porous carbon material DMSO dispersion were performed. According to the normalized transmittance at position z,

In order to explore the OL response intensity of the Au NPs/PC nanocomposites, as a reference, z-scan measurements of porous carbon material DMSO dispersion were performed. According to the normalized transmittance at position z,

(1) $$T=\frac{T\left(i\right)}{T_{0}},$$

where $T\left(i\right)$ is the transmittance of the Au NPs/PC nanocomposites at different positions and $T_{0}$ is the linear transmittance, which is the sample far away from the focus position. At 532 nm, the pulse energy changed from 40 μJ to 160 μJ; the normalized transmittance curves of Au NPs/PC nanocomposites dispersion are given in Figure 6. 

When the sample is far away from the focus, the normalized transmittance of Au NPs/PC nanocomposites is equal to one, corresponding to the linear transmission state. As the sample is close to the focus, the normalized transmittance of the Au NPs/PC nanocomposites decreases rapidly, and the aperture of the normalized transmittance curve of the Au NPs/PC nanocomposites is larger.

**Figure 7:**
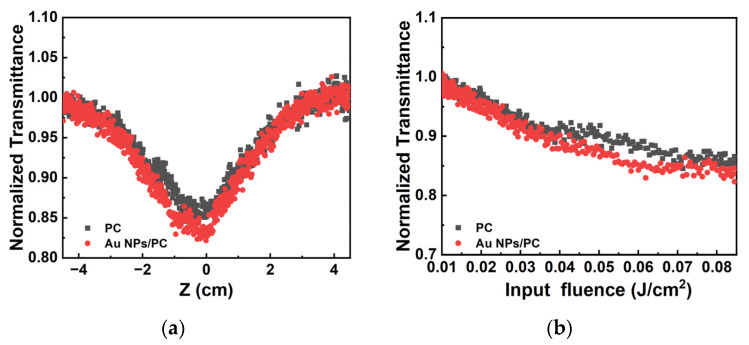
At 532 nm: the contrast curves of Au NPs/PC composites and porous carbon materials (**a**) The normalized transmittance in different z positions and (**b**) normalized transmittance in different incident light power.

In order to study the OL behavior of the Au NPs/PC nanocomposites, as a reference, z-scan measurements of porous carbon DMSO dispersion are performed. At 532 nm, keeping the pulse energy at 100 μJ, the comparison curves of the normalized transmittance of the Au NPs/PC nanocomposites and porous carbon materials are shown in Figure 7. The samples exhibit the typical reversed saturable absorption (RSA). The opening of the transmittance curve of the Au NPs/PC nanocomposites become larger than the porous carbon dispersions in Figure 7a. The results indicate that the Au NPs/PC nanocomposites show the better OL effect at 532 nm. Due to the interaction between the Au nanoparticles and porous carbon materials, the Au NPs/PC nanocomposites show better RSA. According to the plus energy density of the incident light at position z,

(2) $$I\left(z\right)=\frac{I_{0}}{\pi \omega_{0}^{2}\left(z^{2}/z_{0}^{2}\right)},$$

we convert the sample position to the energy density of the incident laser. Among them, $I_{0}$ denotes the energy of the incident light; $\omega_{0}$ is the beam waist radius of the Gaussian beam at the focus position of 190 μm; $z_{0}$ is the Rayleigh length of 1.5 cm; and z denotes the distance from the sample to the focus of the lens (cm). As shown in Figure 7b, the comparison curve of normalized nonlinear transmittance as a function of the incident pulse energy was measured of porous carbon dispersions and Au NPs/PC dispersions. The limiting threshold refers to the input flux as normalized transmittance of the sample drops to 50%. With the incident light power density increased, the Au NPs/PC composite show a lower normalized transmittance at the focus position (z = 0). It is shown that the interaction between porous carbon and Au nanoparticles improves the optical nonlinearity of the composites.

**Figure 8:**
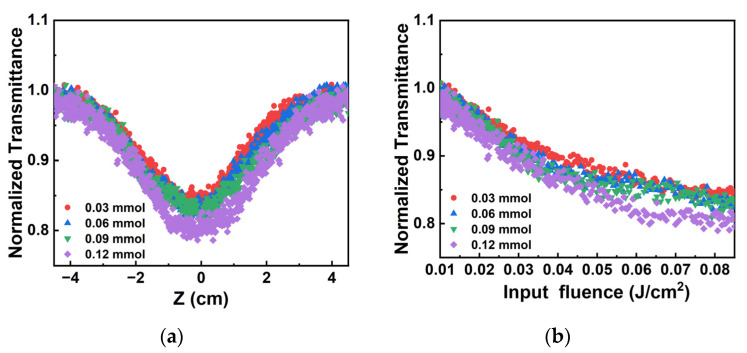
At 532 nm: the contrast curves of different Au^3+^ concentrations of (**a**) the normalized transmittance with the z position changes and (**b**) the normalized transmittance with the incident pulse energy density changes.

In order to study the OL response of different concentrations of Au^3+^, the Au^3+^ concentration was changed to 0.03 mmol, 0.06 mmol, 0.09 mmol and 0.12 mmol. The pulse energy is 100 μJ at 532 nm. As shown in Figure 8a, the opening of the normalized transmittance curve of the Au NPs/PC nanocomposites become larger and the normalized transmittance at the focus position is lower with the higher concentration of Au^3+^. Figure 8b shows the normalized nonlinear transmittance curve in the different incident pulse energy density of Au NPs/PC nanocomposites. With the increase of Au^3+^, the normalized transmittance curve of Au NPs/PC dispersions became lower. Therefore, with the increase in Au^3+^ concentration, the curve of Au NPs/PC nanocomposites shows wider opening and lower transmittance (z = 0). It means that the interaction between large-sized gold nanoparticles and porous carbon show the better OL effect.

In order to measure the nonlinear optical confinement performance of the dispersion, the nonlinear optical confinement coefficient *δ* is defined:

In order to measure the nonlinear optical confinement performance of the dispersion, the nonlinear optical confinement coefficient *δ* is defined:

(3) $$\delta =T_{0}-T_{\min},$$

where $T_{0}$ is the linear transmittance of dispersion without nonlinear optical effects, and $T_{\min}$ is the minimum laser transmittance of dispersion with the nonlinear optical limiting effects. As shown in Table 1, from the OL experimental results of Au NPs/PC composite and porous carbon material dispersion, the best of limiting ability *δ* is 0.122 with the increase in Au^3+^ concentration.

In previous studies, the mechanism of optical limiting effect in porous carbon materials is mainly due to NLS. The nonlinear optical principle of Au nanoparticles is mainly due to NLS caused by plasma and microbubble scattering and nonlinear absorption. NLA is due to effective 2PA, which can be attributed to the generation of free carriers and the existence of defect states. In a high incident light power, the limiting mechanism of Au NPs/PC composites is mainly due to excited-state absorption (ESA) and NLS, resulting in the attenuation of incident light power [19]. Among them, the origin of ESA caused by the free carrier absorption in Au nanoparticles and the increase in defect states caused by the modification of different functional groups in Au NPs/PC composites. The origin of NLS may be derived from the formation of plasma and microbubble scattering centers in nanostructured samples. In addition, the absorption of photogenerated carriers in the conduction band of Au nanoparticles promotes further absorption of the excited state, resulting in a decrease of the normalized transmittance at higher intensities.

## 4. Conclusions

In summary, we prepared Au NPs/PC nanocomposites by the reduction method. Due to the hydrophilic property of the porous carbon, the prepared Au NPs/PC can be monodispersed in solvents and the dispersions in solvents. The influence of reactant concentration on the nonlinear optical response of Au NPs/PC nanocomposites were studied by nanosecond laser z-scan technology. The results indicated that Au NPs/PC nanocomposites have better OL effect than pure porous carbon materials. The OL mechanism is mainly due to the excited-state absorption effect of Au nanoparticles, and the enhanced nonlinear scattering effect is induced by the synergistic effect of Au NPs/PC.

## Figures and Tables

**Table 1:** Comparison of optical limiting properties of Au NPs/PC composites prepared with different Au^3+^ concentrations.

Au^3+^ Concentration (mmol)	$T_{\min}$	*δ*
0	0.578	0.066
0.01	0.548	0.094
0.03	0.541	0.103
0.06	0.53	0.111
0.09	0.526	0.12

## Data Availability

No new data were created or analyzed in this study.
